# Glycosylation Heterogeneity of α1-Acid Glycoprotein: Disease-Associated Glycoform Remodeling, Regulatory Mechanisms, and Clinical Translation

**DOI:** 10.3390/biom16101419

**Published:** 2026-10-01

**Authors:** Gang Liu, Ning Fan, Yating Wang, Weijie Dong

**Affiliations:** College of Basic Medical Sciences, Dalian Medical University, 9-Western Section, Lvshun South Road, Dalian 116044, China; gliu@dmu.edu.cn (G.L.); fanning@dmu.edu.cn (N.F.);

**Keywords:** α1-acid glycoprotein, N-glycosylation, glycan microheterogeneity, site-specific glycosylation, glycan remodeling

## Abstract

α1-Acid glycoprotein (AGP) is a heavily glycosylated acute-phase protein whose N-linked glycan microheterogeneity plays an important regulatory role in pathophysiological processes. This review synthesizes decades of basic and clinical research, focusing on the five conserved N-glycosylation sites of AGP. Specifically, it summarizes disease-specific patterns of glycan remodeling in inflammation, cancer, and autoimmune diseases; discusses the molecular effects through which glycosylation modulates AGP conformation, receptor-binding properties, and downstream signaling pathways; examines disease-associated molecular fingerprints arising from site-specific glycosylation and their relationships with disease onset and progression; and highlights the translational potential of AGP glycosylation for early diagnosis, prognostic assessment, and targeted drug development. Current evidence indicates that most abnormalities in AGP glycosylation are associated with disease, whereas direct causal evidence for the involvement of glycosylation in disease progression remains incomplete. Important limitations also remain: the precise molecular mechanisms regulating AGP glycosylation under different pathological conditions have not been fully elucidated; experimental findings are inconsistent across cohorts and analytical platforms; and standardized analytical systems suitable for clinical application have yet to be established. By integrating the available evidence, this review provides a theoretical framework for advancing the translation of AGP glycosylation features into precision medicine.

## 1. Introduction

α1-Acid glycoprotein (AGP), also known as orosomucoid (ORM), is a major acute-phase protein synthesized predominantly by hepatocytes. Lower levels of AGP are also produced and secreted by extrahepatic tissues, including lung, breast, and colorectal tissues [1,2]. Glycans account for up to approximately 45% of the molecular mass of AGP and confer its characteristic acidity and strongly negative charge, largely because of extensive terminal sialylation [2,3]. Structurally, human AGP contains five N-glycosylation sites and can theoretically generate more than 100,000 glycoform combinations [3]. However, even with a heart-cut two-dimensional nanoLC-CZE-MS platform, only 368 putative glycoforms have thus far been detected at the intact-protein level [4], with the detected glycans being predominantly highly branched, highly sialylated, and multifucosylated, suggesting that AGP glycosylation is subject to precise homeostatic control under physiological conditions. In addition to glycosylation, AGP expression is also subject to coordinated transcriptional regulation by multiple signaling inputs: pro-inflammatory cytokines such as TNF-α and IL-1, together with AGP/EBP (C/EBPβ) transcription factors, participate in the regulatory network governing AGP expression [5,6], collectively shaping its biological functions.

For many years, AGP was regarded in clinical practice primarily as a conventional acute-phase marker of inflammation, whereas its glycosylation heterogeneity, particularly the functional significance of site-specific glycoform remodeling, received comparatively limited attention. In recent years, advances in glycomics have increasingly revealed disease-specific reprogramming of AGP glycoforms and its regulatory relevance to pathological processes. Nevertheless, the patterns of AGP glycoform remodeling across different disease contexts and the associated upstream and downstream regulatory networks remain incompletely understood and, in some cases, controversial. The available evidence also remains fragmented, underscoring the need for systematic synthesis and integration of the findings in this field.

Glycosylation is a major post-translational modification of proteins and plays important roles in fundamental biological processes, including cell communication and signal transduction, by modulating protein conformation and intermolecular interactions [7,8,9]. AGP exhibits extensive structural diversity in its glycans, primarily along three dimensions: glycan branching, reflected by differences in the relative abundance of bi-, tri-, and tetra-antennary structures [10,11,12,13]; sialic acid linkage, particularly α2,3- and α2,6-linked sialylation [5]; and fucosylation, including α1,3-fucosylation [14]. In pathological microenvironments, dysregulated expression of glycosyltransferases and glycosidases may drive AGP glycoform remodeling and may alter multiple biological functions, including immunomodulation, drug binding, and metabolic regulation. These changes may ultimately give rise to molecular features with disease-discriminating potential. In this review, we metaphorically refer to such features as a “glycan code”; this term is not intended to represent an established or standardized concept in the field. Such glycan features may provide potential molecular biomarkers and therapeutic targets for precision diagnosis and treatment.

This review draws on basic and clinical studies of AGP published from 1981 to 2026. Focusing on AGP glycosylation heterogeneity, we examine patterns of site-specific glycan remodeling across disease contexts, the regulatory axis linking glycosylation, protein conformation, and biological function, and receptor-mediated signaling mechanisms through which AGP may contribute to disease progression. We also critically evaluate discordant findings and current methodological and translational challenges and outline priorities for future research. Our aim is to provide an integrated framework for understanding the biological significance of AGP glycosylation and for evaluating its potential translation into diagnostic and therapeutic applications.

Two categories of evidence are considered in this review and are distinguished throughout the manuscript. The first consists of observed, disease-associated glycosylation changes: differences in AGP glycan profiles reported between patient cohorts and control groups, which are predominantly correlative in nature. The second consists of mechanistic evidence: interventional or functional studies demonstrating that a specific glycan feature alters disease-relevant biology, such as receptor binding, signaling activity, or experimentally defined disease endpoints. Most of the available literature on AGP glycosylation in human disease falls into the first category. Unless supported by mechanistic evidence, the findings discussed in Section 3, Section 4 and Section 5 should therefore be interpreted as associations rather than as evidence that the described glycosylation changes directly contribute to disease development or progression.

## 2. Structural Basis and Regulation of AGP N-Glycosylation

### 2.1. Structural Features and Site-Specific Heterogeneity of AGP N-Glycosylation

Mature human AGP comprises 183 amino acid residues, and its five N-glycosylation sites constitute a key structural basis for protein function [15,16,17]. AGP carries predominantly complex-type N-glycans, which are commonly decorated with sialic acid and fucose residues. These glycosylation features not only contribute directly to AGP bioactivity [15,18,19] but also modulate molecular charge distribution and protein conformation, thereby participating in immune recognition, inflammatory regulation, and other biological processes [20]. Mass-spectrometric analyses have revealed extensive diversity among AGP glycoforms and their isomeric forms; combined separation-mass spectrometry platforms achieve effective separation and mass-spectrometric identification of these AGP glycoisomers [21]. High levels of sialylation and extensive glycan branching are prominent structural features of AGP glycans; these physicochemical properties confer a pronounced negative charge and distinctive conformational characteristics on AGP, thereby contributing to the regulation of inflammatory responses and immunomodulatory functions.

**Figure 1:**
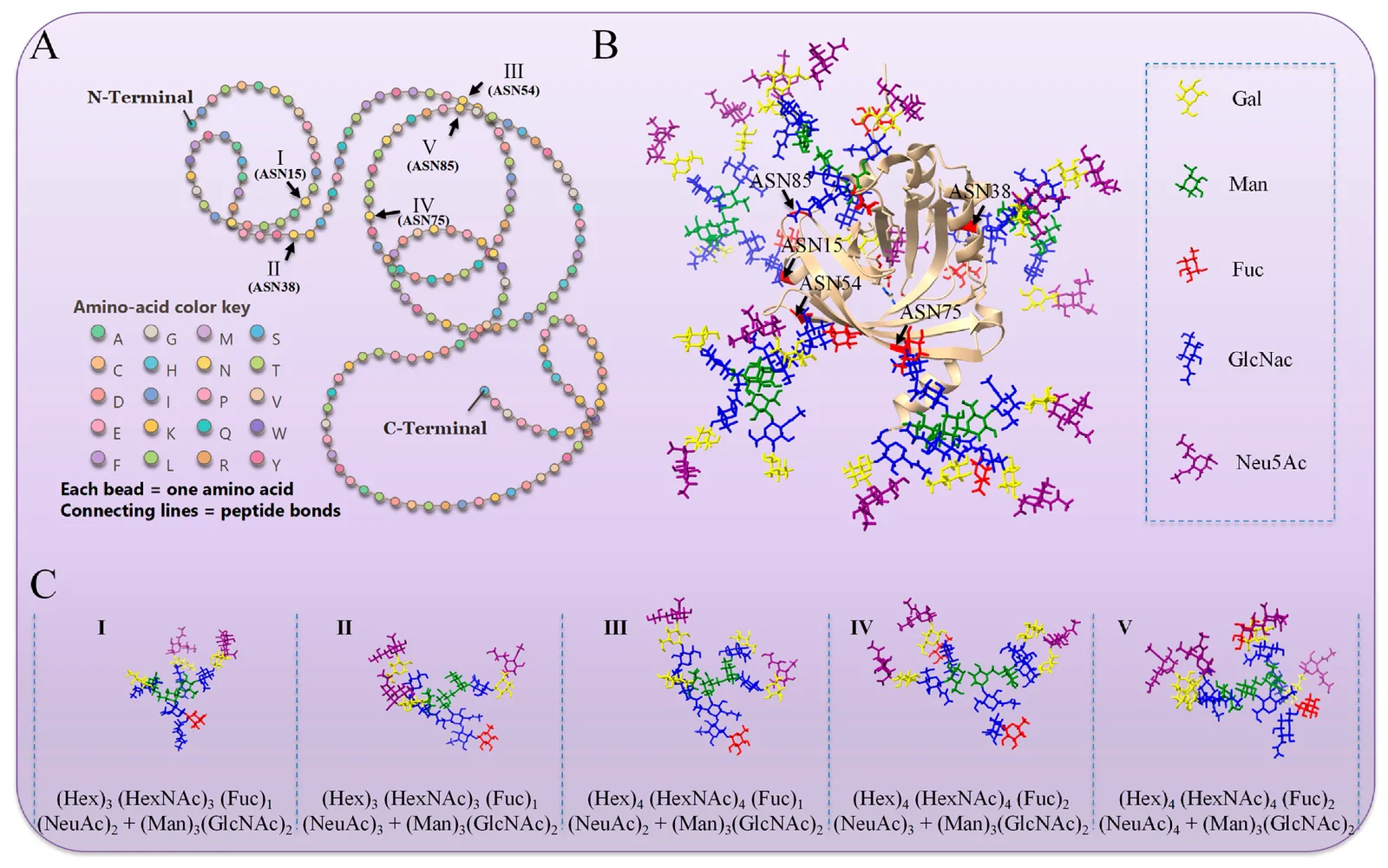
Theoretical three-dimensional models of human α1-acid glycoprotein (AGP) and representative N-glycan structures at its five conserved N-glycosylation sites. (**A**) Topological representation of the AGP primary amino acid sequence, indicating the five conserved N-glycosylation sites (sites I–V) and the N- and C-termini. Colored circles represent individual amino acid residues, and connecting lines represent peptide bonds. (**B**) Three-dimensional model of fully glycosylated AGP, showing the complete protein structure with a representative N-glycan modeled at each of the five conserved N-glycosylation sites. The model illustrates the theoretical conformations of the glycans linked to the corresponding asparagine (Asn) residues. Monosaccharides are color-coded as follows: galactose (Gal, yellow), mannose (Man, green), fucose (Fuc, red), N-acetylglucosamine (GlcNAc, blue), and N-acetylneuraminic acid (Neu5Ac, purple). (**C**) Representative three-dimensional models of the N-glycans at sites I–V, with the complete monosaccharide stoichiometry indicated beneath each structure. Sites I and II contain highly sialylated, monofucosylated tri-antennary N-glycans with different degrees of sialylation, whereas sites III, IV, and V contain highly sialylated, fucosylated tetra-antennary N-glycans; sites IV and V additionally exhibit difucosylated features.

Structural studies indicate that, although the AGP polypeptide scaffold can be modeled computationally, the intrinsic flexibility and microheterogeneity of its glycans make the fully glycosylated protein difficult to resolve directly by crystallography. Small-angle X-ray scattering (SAXS), combined with molecular-dynamics simulations, has enabled the construction of representative three-dimensional computational models of AGP and has provided initial evidence that glycosylation influences molecular motions, thereby providing a model framework for investigating glycan-dependent regulation of protein function [22]. Importantly, the glycans attached at different N-glycosylation sites are intrinsically non-equivalent (Figure 1) and can undergo site-specific remodeling under pathological conditions [15]. This feature indicates that site-level heterogeneity should be taken into account when interpreting the physiological functions of AGP and developing disease biomarkers. High-throughput LC-MS enables detailed characterization of site-specific AGP glycosylation patterns in population samples and provides an important analytical basis for elucidating their roles in health and disease [15]. To systematically compare branching, sialylation and fucosylation features across these five sites, Table 1 summarizes the structural characteristics and glycan modification patterns of the five conserved N-glycosylation sites of AGP, allowing direct comparison of site-level differences.

As shown in Table 1, AGP glycosylation patterns can be classified into two groups according to the degree of glycan branching: low-branching sites (I and II) and high-branching sites (III, IV, and V). Sites I (Asn-15) and II (Asn-38) are dominated by tri-antennary complex-type glycoforms, principally TriS3, with smaller proportions of bi-antennary structures. Their sialylation is mainly distributed within the S2–S3 range, whereas F1 fucosylation represents only a minor modification. By contrast, the high-branching sites (III, IV, and V) are dominated by tetra-antennary complex-type glycoforms, principally TetraS3 and TetraS4, and exhibit substantially greater branching complexity than sites I and II, while also containing smaller proportions of tri- and bi-antennary glycans. Site IV (Asn-75) exhibits the greatest glycoform diversity. In addition to the predominant tetra-antennary glycoforms, glycans bearing additional N-acetyllactosamine (Gal–GlcNAc) units are also detected. In the source study these glycans are designated penta- and hexa-antennary, however, as noted in that study, the prefixes denote the number of LacNAc units rather than the number of antennae. The additional Gal–GlcNAc units may therefore reflect either an increased number of antennae or poly-LacNAc extension of the existing antennae, and these two structural alternatives cannot be distinguished from the mass-spectrometric data reported in [11] (see also the note to Table 1). Sialylation at this site can reach S4, and both F1- and F2-fucosylated species are present. Site V (Asn-85) is overwhelmingly dominated by TetraS4 and shows the highest abundance of tetra-antennary, tetrasialylated glycans among the five sites. Minor glycans bearing additional Gal–GlcNAc units are also present and are subject to the same interpretational caveat as for site IV, together with both F1- and F2-fucosylated forms.

Available evidence indicates that human AGP exhibits pronounced site-specific N-glycosylation. This site specificity can be explained, at least in part, by the three-dimensional fold of the protein: upon folding, each N-glycosylation site occupies a distinct local microenvironment that modulates substrate accessibility to branching glycosyltransferases, thereby shaping the branching pattern and terminal elaboration of glycans at individual sites [22,23,24]. Fully glycosylated three-dimensional models derived from SAXS and molecular-dynamics simulations visualize these spatial differences, while crystallographic and biochemical studies of glycosyltransferases further provide a general molecular basis for steric control of glycan biosynthesis. The regions surrounding sites I and II are relatively sterically constrained, which can limit the formation of highly branched glycans. By contrast, the local conformational environments of canonical high-branching sites such as IV and V are more permissive to glycan extension and multibranch formation, resulting in marked site-dependent differences in glycan abundance and structure. This site-dependent glycosylation pattern constitutes a major structural basis for AGP glycoform heterogeneity and may contribute to the regulation of biological processes such as neutrophil adhesion and inflammatory responses. Under pathological conditions, AGP glycans undergo characteristic remodeling in branching complexity and sialylation. The recurrent AGP glycosylation abnormalities reported in cancer, chronic inflammation, and related pathological conditions support the potential of AGP glycoforms as candidate clinical biomarkers.

Taken together, glycosylation endows AGP with multiple layers of structural heterogeneity and functional plasticity and represents a central focus for understanding AGP physiology and elucidating the molecular mechanisms through which AGP may contribute to disease initiation and progression.

### 2.2. Biosynthesis and Regulation of AGP N-Glycosylation

**Figure 2:**
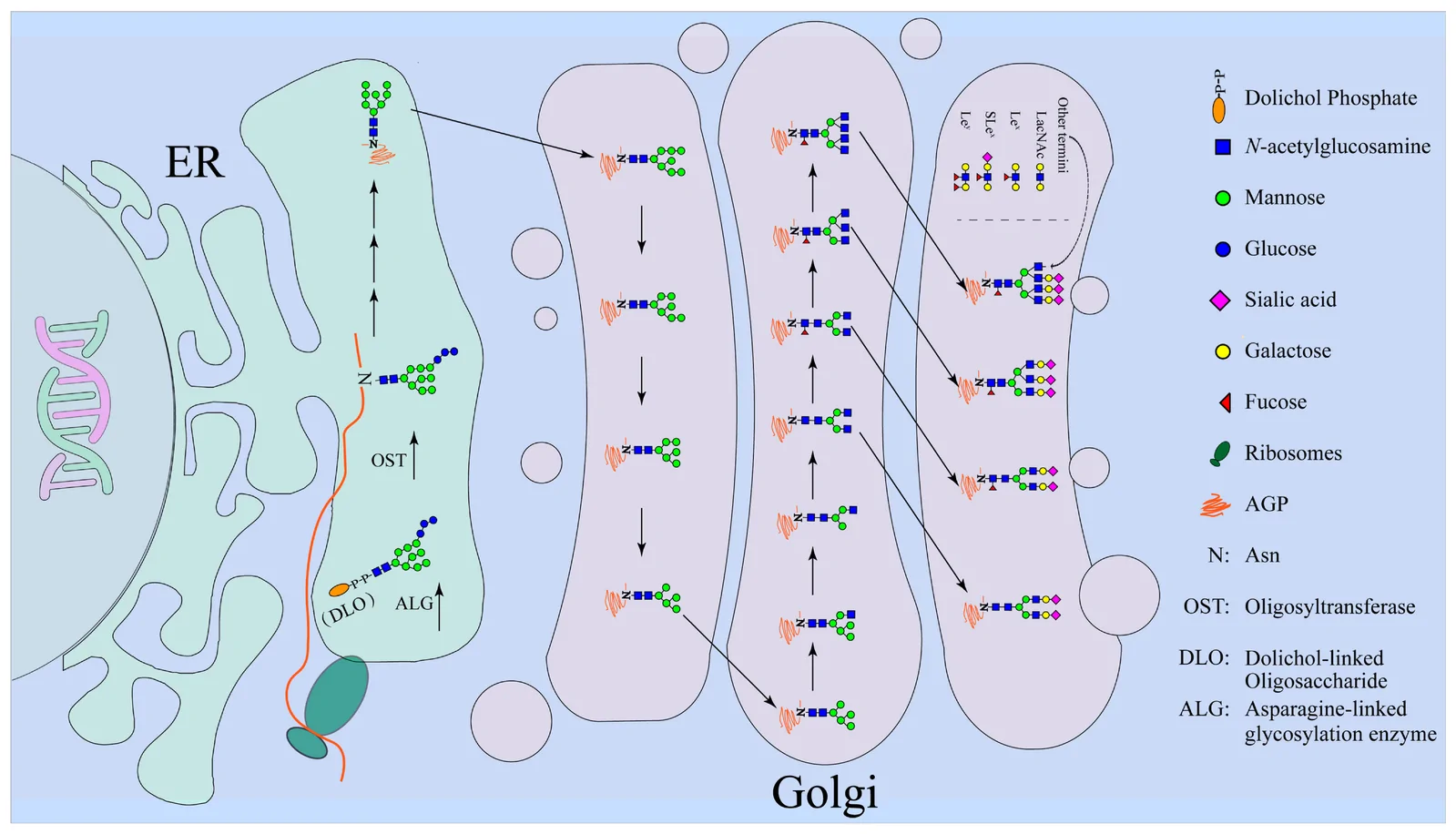
Overview of the conserved mammalian N-glycan biosynthetic pathway as it applies to AGP. A dolichol-linked oligosaccharide (DLO) precursor (Glc_3_Man_9_GlcNAc_2_) is assembled on dolichol phosphate at the ER membrane by the ALG (asparagine-linked glycosylation) enzyme system and, after translocation into the ER lumen, is transferred en bloc by oligosaccharyltransferase (OST) to Asn residues within Asn-X-Ser/Thr sequons of the nascent polypeptide. Subsequent trimming by glycosidases and stepwise extension by glycosyltransferases in the ER and in the cis-, medial-, and trans-Golgi compartments generate branched structures and terminal moieties such as sialic acid and fucose, yielding mature N-glycans of the oligomannose, hybrid, and complex types. The pathway description follows [26,30]; the scheme depicts the general biosynthetic framework and does not imply that every step shown has been experimentally demonstrated for AGP specifically. Abbreviations: OST, oligosaccharyltransferase; DLO, dolichol-linked oligosaccharide; ALG, asparagine-linked glycosylation enzymes; ER, endoplasmic reticulum. Adapted from Liu et al. [31], licensed under CC BY 4.0.

The general pathway of N-glycan biosynthesis is a dynamic, compartmentalized, and stepwise process that proceeds through the endoplasmic reticulum (ER) and Golgi apparatus [25,26]. In human AGP, a model glycoprotein for N-glycosylation studies, the dynamic balance between the catalytic activities of Golgi glycosyltransferases and glycosidases is thought to shape higher-order glycan structures, including glycan branching and terminal modifications, and may therefore represent a major regulatory mechanism underlying its extensive glycoform heterogeneity. AGP glycoforms exhibit pronounced heterogeneity, with glycan branching, sialylation, and fucosylation varying according to physiological and pathological states [10,20,27]. However, direct evidence demonstrating that this enzymatic balance governs the entire AGP glycan-processing pathway is currently lacking. Golgi-resident fucosyltransferases and sialyltransferases are key enzymes involved in terminal glycan maturation. Fucosyltransferases selectively catalyze the incorporation of fucose residues and thereby regulate the overall extent of AGP fucosylation, whereas sialyltransferases determine the linkage types of terminal sialic acid residues. These two classes of glycosyltransferases can act sequentially in the Golgi to elaborate terminal glycan structures [28]. The sialylated and fucosylated complex glycans displayed on mature AGP can influence protein conformational dynamics and contribute to selectin recognition, thereby modulating inflammation-related biological responses [10]. Meanwhile, ER- and Golgi-resident glycosidases, including α-glucosidases and α-mannosidases, participate in glycan quality control by sequentially trimming nascent glycan precursors, removing inappropriate monosaccharide residues, and hydrolyzing excess terminal sugar residues. This tightly regulated processing promotes orderly glycan assembly, limits the accumulation of aberrant glycan structures, and ultimately contributes to the maintenance of cellular glycan homeostasis. The compartmentalized glycosyltransferase–glycosidase processing system of the ER and Golgi constitutes a major biosynthetic pathway responsible for generating glycan diversity on secreted proteins [26,29,30]. This conserved processing pathway is also applicable to AGP, and the overall process is illustrated in Figure 2. As a typical secreted glycoprotein undergoing this multistep enzymatic processing pathway, AGP contains five N-glycosylation sites and consequently generates a broad repertoire of site-specific glycoforms [10,26,29].

Beyond the constitutive processing mediated by ER- and Golgi-resident glycan-modifying enzymes, extracellular inflammatory signals can alter the transcription of these enzymes and thereby indirectly remodel AGP glycosylation. Pro-inflammatory cytokines, exemplified by IL-1β, have been associated with AGP glycoform remodeling in pathological microenvironments. Inflammatory stimulation has been reported to increase the transcription of sialyltransferases and fucosyltransferases, including ST3Gal IV and FUT VI, and to increase the abundance of complex glycans such as sialyl Lewis X (sLe^X^) on AGP [32,33]. These changes may alter the molecular recognition properties of AGP and thereby modulate downstream inflammation-related biological responses. Collectively, these observations are consistent with inflammation-driven remodeling of AGP terminal glycosylation, although the complete causal sequence linking glycosyltransferase induction to defined AGP glycoforms has not been directly established in all experimental settings. Based on shared inflammatory signaling pathways, other pro-inflammatory cytokines, including IL-6 and TNF-α, have been proposed to exert similar regulatory effects; however, direct experimental evidence remains limited.

Modern high-resolution mass-spectrometric platforms provide essential analytical tools for dissecting this multilayered and highly heterogeneous glycosylation-regulatory process. In particular, online nanoflow liquid chromatography-capillary zone electrophoresis-mass spectrometry (nanoLC-CZE-MS) combines two orthogonal separation dimensions and enables highly sensitive characterization of AGP glycosylation.

## 3. Disease-Associated Patterns of AGP Glycosylation Remodeling

### 3.1. AGP Glycosylation Remodeling in Inflammatory Diseases

Inflammatory microenvironments can induce characteristic remodeling of AGP glycosylation, and the patterns of AGP glycoform changes differ markedly between acute inflammation and non-autoimmune chronic inflammation [34].

#### 3.1.1. Characteristic AGP Glycoform Remodeling in Acute Inflammation

During acute inflammatory conditions, such as bacterial infection and tissue injury, pro-inflammatory cytokines including IL-6, TNF-α, and IL-1β are released [35]. Studies using primary human hepatocytes in vitro and animal models have shown that IL-1 and IL-6 can alter the hepatocellular processing of AGP glycans [36], resulting in a characteristic circulating AGP glycosylation profile marked by an increased proportion of bi-antennary glycans, reduced tri- and tetra-antennary branching, and increased α1,3-fucosylation [37].

From an immunoregulatory perspective, hepatic AGP synthesized during acute inflammation can undergo glycan remodeling accompanied by a marked increase in α2,6-linked sialylation [38]. Terminal sialic acids have been shown to inhibit excessive activation of the classical complement pathway by sterically shielding target molecules from C1q binding [39]. On this basis, we propose that increased α2,6-linked sialylation of AGP may similarly interfere with C1q–AGP interactions and thereby attenuate aberrant complement activation. Sialylation is also an important structural requirement for ligand recognition by Siglec-9 [40]. Accordingly, highly α2,6-sialylated AGP may retain the capacity to interact with myeloid Siglec-9 and engage inhibitory Siglec-family signaling, thereby contributing to the restraint of inflammatory amplification and excessive immune activation.

#### 3.1.2. AGP Glycosylation Remodeling in Non-Autoimmune Chronic Inflammation

In non-autoimmune chronic inflammatory conditions driven by persistent infection or sustained tissue injury, such as chronic infections and chronic nonspecific tissue damage, fluctuations in AGP glycosylation are closely associated with disease activity. Glycoform characteristics can provide a quantitative readout of persistent inflammatory burden and therefore have potential as candidate biomarkers for longitudinal monitoring of chronic inflammation [41]. Importantly, AGP glycosylation remodeling is not specific to metabolic disease. In patients with type 2 diabetes mellitus, AGP glycosylation alterations appear to be largely associated with concomitant chronic inflammation rather than being directly driven by metabolic dysregulation itself [14]. Obesity and adiposity represent important contextual variables when interpreting AGP glycosylation in metabolic and inflammatory diseases. In 803 individuals from the Croatian island of Korčula, site-specific LC–MS analysis identified associations between AGP N-glycosylation and BMI, as well as other biochemical and physiological traits, including glucose and fibrinogen [42]. These findings support the inclusion of BMI as a covariate in biomarker analyses, although this cross-sectional study cannot establish whether inflammation mediates the observed association. Earlier work based on released-glycan profiling by Higai et al. [14] could not fully rule out metabolic influences on AGP glycosylation. Complementing these observations, a more recent glycopeptide-based analysis reported site-dependent differences in AGP glycosylation in individuals with a history of critical-illness-related hyperglycemia and an elevated risk of type 2 diabetes [15]. Taken together, the interpretation of AGP glycosylation data should account for adiposity, inflammatory status, glycemic traits, and differences in analytical resolution among experimental approaches. Notably, AGP concentration and AGP glycosylation are distinct measurements; neither can substitute for the other when evaluating biomarker performance [15].

### 3.2. AGP Glycosylation Remodeling in Cancer

#### 3.2.1. Characteristic AGP Glycoform Patterns Across Cancer Types

Glycosylation remodeling of circulating AGP is widely observed in patients with cancer. Three major features recur across tumor types: increased sialylation, remodeling of glycan branching, and dysregulated fucosylation, although the detailed glycan signatures differ among cancers. In lung cancer, serum α1,3-fucosylated AGP (fAGP) increases progressively with disease progression, and its abundance has been associated with response to immunotherapy and long-term survival [43]. In breast cancer, AGP fucosylation, high-mannose glycans, and sialylation show tumor-stage-dependent variation; characteristic glycoform alterations can be detected at early stages and may therefore have potential value for adjunctive early diagnosis [44]. In hepatocellular carcinoma (HCC), a distinctive AGP glycoform carrying multifucosylated tetra-antennary N-glycans has been reported. This glycoform can distinguish patients with HCC from healthy individuals and patients with cirrhosis and may therefore support disease stratification [45]. In malignant melanoma, selected fucosylated AGP glycans are increased, whereas non-fucosylated glycans are decreased, accompanied by an increased proportion of α2,6-linked sialic acid. This glycosylation profile may have potential as a serum biomarker to support clinical diagnosis of malignant melanoma [46].

Although aberrant AGP glycosylation is observed across multiple cancer types, the detailed structural alterations in its glycans are highly tumor-type dependent [43,44,45,46]. Colorectal cancer, a common gastrointestinal malignancy, has been investigated using relatively comprehensive quantitative glycomic datasets, allowing detailed characterization of AGP glycosylation remodeling [47]. Table 2 summarizes and quantitatively compares the relative abundances of plasma AGP N-glycans with different antennarity and fucosylation states between patients with colorectal cancer and healthy controls.

#### 3.2.2. Potential Mechanisms Linking the Tumor Microenvironment to AGP Glycosylation Remodeling

Tumor-associated alterations in AGP glycosylation do not represent an entirely distinct regulatory mechanism; rather, they can be viewed as perturbations of the physiological N-glycan-processing pathway described in Section 2.2, superimposed on tumor-associated shifts in glycan modification. Dysregulated glycosyltransferase expression arising from interactions between tumor cells and the extracellular microenvironment may contribute to this pathological remodeling. The available mechanisms can be considered at four partly overlapping levels:

(1) Tumor-associated remodeling of glycosyltransferase expression. ST6Gal-I is upregulated in multiple epithelial tumors, whereas members of the fucosyltransferase (FUT) family show tumor-dependent increases or decreases within malignant lesions. More directly relevant to AGP, hepatic FUT6 has been reported to mediate α1,3-fucosylation of AGP, and increased fucosylated AGP has been observed in patients with cancer [45,48,49,50]. These findings support a link between altered fucosyltransferase activity and tumor-associated AGP glycosylation, although the complete causal pathway from tumor-associated signaling to hepatic AGP glycoform remodeling remains to be defined.
(2) Transcriptional regulation of glycosyltransferases through inflammatory signaling and epigenetic mechanisms. In tumor cells, microenvironmental inflammatory signals can induce aberrant glycosyltransferase expression. Cytokine-induced NF-κB and STAT3 signaling can activate the transcription of glycosyltransferase genes [51]. In parallel, epigenetic mechanisms, including DNA methylation, can reconfigure transcriptional programs governing glycosylation-related enzymes and thereby amplify aberrant cellular glycosylation phenotypes [52,53]. These mechanisms provide a molecular basis for inflammation-associated remodeling of cellular glycosylation, although their direct contribution to hepatocyte-derived circulating AGP glycoforms remains incompletely established.
(3) Amplification by multiple tumor-microenvironmental factors. Microenvironmental signals can act as upstream stimuli that further amplify dysregulated endogenous glycosylation pathways. Hypoxia can enrich for tumor cells with high ST6Gal-I expression, whereas ST6Gal-I can in turn enhance HIF-1α signaling, forming a positive-feedback relationship between hypoxic signaling and glycosyltransferase activity [54]. IL-1β and IL-6 can activate NF-κB and STAT3 through paracrine signaling and promote ST6GAL1 transcription and ST6Gal-I expression [51]. ST6Gal-I also modifies N-glycans on substrates such as TNFR1 and thereby influences apoptotic and survival signaling [55]. These findings demonstrate how the tumor microenvironment can alter cellular sialylation; however, whether increased tumor-associated ST6Gal-I directly accounts for altered sialylation of circulating AGP remains to be established.
(4) Potential immune consequences and feedback involving AGP glycosylation. AGP has been reported to engage CD14/TLR4-associated signaling in macrophages and to influence STAT1/STAT3 signaling, PD-L1 expression, and IL-6 responses [56]. Separately, sLe^X^-bearing AGP can competitively interact with E-selectin and may reduce E-selectin-mediated leukocyte adhesion [57]. Together, these observations suggest potential links among AGP, altered glycosylation, and the tumor immune microenvironment. However, whether these effects are specifically mediated by tumor-associated AGP glycoforms, and whether they constitute a self-sustaining positive-feedback loop that promotes persistent AGP expression, remain to be directly demonstrated.

#### 3.2.3. Immune Effects and Translational Potential of Tumor-Associated AGP Glycosylation

Tumor-associated glycosylation abnormalities in glycoproteins can interfere with immune-checkpoint function and suppress immune-cell cytotoxicity, thereby contributing to tumor immune evasion [58]. AGP has been reported to interact with membrane receptors on macrophages, suppress aspects of T-cell activation, proliferation, and cytotoxic function, and promote macrophage polarization toward an immunosuppressive M2-like phenotype [56,59]. However, the extent to which these immunoregulatory effects are specifically mediated or enhanced by tumor-associated AGP glycoforms remains incompletely defined. Altered AGP glycosylation may also influence the immune state of the tumor microenvironment through changes in receptor recognition and cytokine-associated signaling, potentially further promoting immune escape. Based on these immunoregulatory properties and disease-associated glycoform changes, AGP glycosylation has emerged as a candidate biomarker axis and an important mechanistic research focus in cancer, with potential relevance to early detection and therapeutic development [41,60]. Further elucidation of the molecular role of AGP glycosylation within tumor-immune regulatory networks, together with integrated glycomic and multi-omic profiling and validation in large clinical cohorts, may support earlier cancer detection, molecular stratification, and individualized immunotherapy. Such studies will be essential for determining whether AGP glycoform signatures can be translated into clinically useful diagnostic or therapeutic strategies.

### 3.3. AGP Glycosylation Remodeling in Autoimmune Diseases

#### 3.3.1. Structural Features of AGP Glycans and the Basis of Immune Recognition

Altered AGP glycosylation has been reported in autoimmune diseases, with rheumatoid arthritis (RA) representing the best-characterized example. Changes in glycan structure may alter the biological functions of AGP and may also provide candidate biomarkers for disease diagnosis and monitoring of disease activity [15,41,61]. AGP carries predominantly complex-type N-glycans containing Galβ1-3GlcNAc and Galβ1-4GlcNAc motifs and terminal sialylation, with variation in the relative proportions of α2,3- and α2,6-linked sialic acids. These structural features influence protein stability, immunoregulatory activity, and intermolecular interactions [3]. The intrinsic heterogeneity of AGP glycans has also been implicated in interactions with immune-cell surface receptors, including CD14 and CCR5 [27,35,62]. However, glycoform-resolved binding affinities and their specific relevance to autoimmune disease remain incompletely defined.

#### 3.3.2. AGP Glycoform Remodeling in Rheumatoid Arthritis

RA is a prototypic autoimmune disease characterized by distinct remodeling of the AGP glycosylation profile. Two major glycosylation changes have been reported: a marked increase in glycan branching [63] and an overall increase in fucosylation [57,64,65]. Notably, the magnitude of increased AGP fucosylation has been reported to correlate positively with inflammatory severity in RA [65]. AGP fucosylation also exhibits a pronounced sex-related difference [64]. In healthy individuals, baseline AGP fucosylation is significantly higher in men than in women, and this sex-related difference is also observed in patients with recent-onset RA, with male patients showing significantly higher AGP fucosylation than female patients. A relationship between AGP fucosylation and the 28-joint Disease Activity Score (DAS28) has been reported only in male patients, suggesting its potential value as a supportive marker of disease activity in men; no corresponding association has been observed in female patients. In addition, AGP from patients with RA shows increased expression of the sLe^X^ glycan epitope. This glycan feature can modulate the interaction of AGP with E-selectin and thereby influence leukocyte adhesion to vascular endothelium under inflammatory conditions [10,27,57]. The molecular consequences of the sLe^X^–E-selectin interaction are discussed in detail in Section 4.2 and are therefore not repeated here. Glycoform composition in RA also varies with disease duration and activity. Using Con A affinoimmunoelectrophoresis, increased AGP–Con A reactivity was observed in patients with very recent RA of no more than three months’ duration, whereas normal or low reactivity was observed in patients with early RA of longer duration. In established RA, variants with low Con A affinity predominated. In both early and established RA, AGP–Con A reactivity decreased with increasing disease activity [66]. Dynamic remodeling of AGP glycans has been associated with the intensity of inflammatory responses and may thus serve as a molecular indicator of changing inflammatory burden [67].

#### 3.3.3. Potential Molecular Mechanisms Linking AGP Glycosylation to Autoimmune Dysregulation

At the molecular level, remodeling of AGP glycosylation has been associated with autoimmune inflammation and may contribute to disease-related immune regulation. Disease-associated shifts in sialylation and fucosylation can modulate the binding of AGP to vascular endothelial E-selectin [3,57,68]. One hypothesis is that aberrantly glycosylated AGP may activate central inflammatory pathways such as NF-κB and JAK-STAT and thereby amplify autoimmune responses [27,69]; however, this proposition has not yet been supported by direct in vivo functional evidence. More generally, N-glycosylation of membrane proteins can stabilize pro-inflammatory receptors at the cell surface, strengthen downstream signaling, and promote amplification of systemic inflammatory cascades [70]. These broader observations provide a mechanistic framework but do not demonstrate an AGP-specific pathway. Glycosyltransferase modulation to reshape AGP glycosylation should therefore be regarded as a promising but still exploratory therapeutic direction in autoimmune disease.

#### 3.3.4. Clinical Relevance of AGP Glycosylation in Autoimmune Diseases

Cohort studies have documented characteristic remodeling of plasma AGP fucosylation in patients with RA. Baseline AGP fucosylation levels were significantly associated with C-reactive protein (CRP) and TNF levels but showed no significant association with baseline DAS28. In a 54-week longitudinal follow-up of patients receiving infliximab, dynamic changes in AGP fucosylation tracked clinical treatment response, suggesting that AGP fucosylation may serve as a candidate biomarker for disease assessment and therapeutic-response monitoring in RA [65].

### 3.4. Comparative Summary of AGP Glycosylation Features Across Pathological Conditions

Across acute inflammation, non-autoimmune chronic inflammation, cancer, and autoimmune disease, AGP exhibits distinct glycosylation profiles, providing a potential molecular basis for disease differentiation using AGP glycomic signatures. To facilitate comparison of the major AGP glycosylation features across these pathological conditions, the key findings described above are summarized in Table 3, with the corresponding primary references provided directly in Table 3.

A methodological distinction should be noted when comparing these findings. Much of the earlier disease-associated AGP glycomics literature reports released-glycan profiles, whereas site-resolved glycopeptide data have only recently become available. Because AGP glycans are distributed across five N-glycosylation sites with distinct glycan repertoires, an alteration observed at the released-glycan or intact-glycoform level reflects the summed contribution of all five sites and cannot be attributed to a particular site without site-specific analysis. Among the studies summarized in Table 3, only [37] provides site-resolved evidence obtained in a disease-associated context; conclusions regarding site-specific remodeling are therefore drawn only where such evidence is available.

## 4. Functional Consequences of AGP Glycosylation Remodeling

### 4.1. Glycosylation-Dependent Modulation of AGP Conformation and Ligand Recognition

Glycosylation can alter the physicochemical properties of AGP, including charge distribution and conformational dynamics, and may thereby influence its three-dimensional conformation and receptor–ligand recognition. Glycan microheterogeneity is therefore an important molecular basis for the diverse immunoregulatory functions of AGP [35,71]. Fine structural features of AGP glycans, including branching and terminal modifications, can influence protein function through two principal physicochemical mechanisms. First, electrostatic interactions: the strong negative charge of terminal sialic acids can contribute directly to ligand binding by altering electrostatic attraction or repulsion between AGP and molecules such as complement proteins and lectins [35,71]. The general role of terminal sialylation in modulating intermolecular electrostatic interactions has been supported by in vitro protein models [72,73], although the AGP-specific mechanism remains to be fully defined. Second, steric effects: structural features such as glycan branching and the extent of sialylation can create a steric barrier on the protein surface [74]. Such steric shielding may alter the accessibility and binding kinetics of AGP toward immune receptors and thereby modulate its immunoregulatory activity [27,71,74]. These glycosylation-dependent conformational effects may have important pathological consequences. During inflammation, AGP undergoes sialylation remodeling. While electrostatic forces modulate many AGP–ligand interactions, AGP binding to LPS operates through a distinct mechanism. Although intact sialylation confers a negatively charged surface to AGP, simple electrostatic attraction cannot drive AGP-LPS binding given that LPS also carries abundant negative charges derived from phosphate and Kdo moieties. Instead, molecular-modelling data suggest that surface sialylated N-glycans near the AGP ligand-binding pocket entrance stabilize AGP-LPS complexes via polar carbohydrate-carbohydrate interactions between AGP N-glycans and the LPS core oligosaccharide and O-antigen, enabling AGP to bind and neutralize LPS and thereby attenuate LPS-driven proinflammatory signaling [75]. This effect appears to be conditional and depends strongly on AGP’s local concentration [75]. Clinical lectin-array and mass-spectrometric glycomic studies have demonstrated significantly increased levels of highly fucosylated, multifucosylated tetra-antennary AGP glycoforms in the sera of patients with liver cancer [45,76,77]. Based on general physicochemical principles of glycoproteins, it has been hypothesized that these characteristic glycan modifications may remodel AGP conformation and thereby contribute to the regulation of malignant phenotypes such as liver cancer cell adhesion and invasion. However, the specific molecular mechanisms underlying this proposed pathway have not yet been directly validated by functional studies in cellular or animal models.

### 4.2. Glycoform-Dependent Receptor Recognition and Downstream Signaling

The interaction of AGP with target-cell membrane receptors is strongly dependent on its glycan-remodeling state. Glycosylation features such as sialylation and fucosylation jointly determine the receptor-binding capacity of AGP and constitute a central molecular basis for AGP–receptor interactions [78,79]. Different AGP glycoforms exhibit distinct recognition preferences toward cell-surface lectin receptors and may thereby selectively modulate multiple downstream inflammation-related immune pathways [18]. Highly sialylated AGP specifically binds Siglec-5 on neutrophils through its terminal sialic acid residues and, following L-selectin priming, can trigger intracellular Ca^2+^ signaling in neutrophils [1,78]. By contrast, α1,3-fucosylation at the termini of AGP N-glycans contributes to the formation of the sLe^X^ epitope on appropriately sialylated glycan termini. Increased sLe^X^ expression alters AGP glycan architecture, weakens the interaction of AGP with neutrophil Siglec-5/14, and suppresses AGP-mediated Ca^2+^ mobilization, indicating that this glycan-remodeling pathway participates in the regulation of leukocyte inflammatory responses [80]. Desialylated AGP (AsAGP), which exposes terminal galactose residues on its glycans, specifically binds the hepatocyte asialoglycoprotein receptor (ASGPR), is taken up by hepatocytes through receptor-mediated endocytosis, and is subsequently degraded in lysosomes [81,82]. Clinical cohort studies have shown that serum AsAGP levels increase significantly with fibrosis progression in patients with cirrhosis or chronic hepatitis B-associated liver injury [83,84]. A hypothesis proposed in the review literature suggests that chronic liver injury impairs hepatocellular ASGPR function, thereby reducing hepatic clearance of AsAGP and leading to its accumulation in the peripheral circulation. However, the specific inference that ASGPR expression is downregulated is derived from review-level synthesis, and direct supporting evidence from original in vitro or in vivo studies is currently lacking [2].

Under inflammatory stimulation, the liver synthesizes and releases circulating AGP bearing the sLe^X^ glycan epitope. This soluble glycoprotein can competitively bind E-selectin on activated endothelial cells, thereby reducing leukocyte–endothelial adhesion and, in principle, inhibiting transendothelial leukocyte extravasation [5,85].

Taken together, AGP glycan microheterogeneity is closely associated with its regulatory functions in inflammation and liver injury. Different AGP glycoforms can act as specific ligands for distinct cell-surface receptors and thereby elicit differential downstream signaling responses, forming a multilayered molecular regulatory network involved in multiple disease-associated pathological processes [5,71,79].

### 4.3. Functional Significance of Site-Specific AGP Glycosylation

As a heavily glycosylated acute-phase protein, AGP relies on site-specific glycosylation as a key structural basis for fine functional regulation. Hydrophilic interaction liquid chromatography (HILIC), combined with reversed-phase chromatography, enables efficient enrichment and separation of AGP glycopeptides and allows confident assignment of N-glycosylation sites and their heterogeneous glycoforms [86,87]. For example, AGP typically contains five conserved N-glycosylation sites, and fucosylation and sialylation exhibit pronounced site-specific distribution patterns across these sites [21,86,87]. Under disease conditions, AGP glycosylation can undergo substantial site-specific reprogramming. Targeted multiple-reaction monitoring (MRM) mass spectrometry has demonstrated ovarian cancer-associated alterations in fucosylation at specific N-glycosylation sites of serum AGP. These site-specific glycopeptide features are significantly associated with advanced epithelial ovarian cancer and have potential as candidate serum diagnostic biomarkers for ovarian cancer [88]. At the functional level, although the underlying regulatory mechanisms remain to be validated, functional specialization among different glycosylation sites has been proposed: fucosylation changes at some sites appear to be involved primarily in inflammatory regulation [89], whereas sialylation-related glycoforms may contribute to the modulation of drug binding and transport [90].

When considered together with the site-specific structural features summarized in Table 1, AGP exhibits a distinct pattern of site-specific glycosylation regulation. Glycosylation at sites I and II is relatively conserved, shows limited interindividual variation among healthy individuals, and generally does not form tetra-antennary structures. Site I shows a specific increase in the proportion of bi-antennary structures during acute inflammation, whereas the glycan-branching pattern at site II exhibits no marked change during either acute or chronic inflammation. By contrast, sites III, IV, and V are characterized mainly by inflammation-associated redistribution of glycan branching and increased α1,3-fucosylation, and these changes are closely associated with both acute and chronic inflammatory processes [37]. This site-specific regulatory pattern provides candidate molecular targets for precise modulation of AGP function and the development of disease-targeted therapeutics.

## 5. Clinical Translation of AGP Glycosylation: Opportunities, Challenges, and Future Directions

### 5.1. AGP Glycoforms as Candidate Diagnostic and Prognostic Biomarkers

AGP glycosylation patterns exhibit pronounced disease specificity and represent multipurpose biomarkers with substantial translational potential, with possible applications in early disease screening, monitoring of inflammatory activity, and prognostic stratification. Dynamic changes in AGP glycoforms not only provide objective quantitative indicators of systemic inflammatory burden but may also reflect molecular trajectories accompanying disease onset and progression. With highly sensitive glycomic technologies, disease-specific AGP glycosylation features can be precisely captured, potentially enabling three major clinical applications: (1) adjunctive diagnosis at very early stages of disease; (2) longitudinal monitoring of disease course; and (3) objective assessment of therapeutic response and long-term prognosis, thereby providing a molecular basis for individualized diagnostic and therapeutic strategies. At present, the clinical translation of AGP glycosylation remains at an early stage. With continued advances in glycomic technologies and the progressive establishment of standardized glycosylation databases, the clinical value of AGP glycosylation as a next-generation diagnostic and prognostic tool is expected to become increasingly apparent.

### 5.2. Therapeutic Potential of Targeting Aberrant AGP Glycosylation

Aberrant AGP glycosylation has been linked to tumor progression and inflammatory diseases, suggesting its potential value as a conceptual therapeutic target. However, the causal relationship between AGP glycoforms and disease pathogenesis remains unestablished, as current evidence is predominantly correlative rather than interventional. Accordingly, the strategies outlined below should be regarded as a hypothetical framework for future investigation rather than established therapeutic opportunities. These strategies can be broadly divided into two categories: molecular blocking, which employs small-molecule inhibitors or monoclonal antibodies to interrupt disease-relevant glycosylation events and their downstream effector interactions; and enzymatic activity modulation, which targets glycosyltransferases or glycosidases to restore physiological glycosylation patterns. The principal obstacle to both approaches is selectivity. Because most glycosylation enzymes possess a broad substrate repertoire, systemic modulation risks global perturbations of glycosylation-dependent biological processes. Achieving AGP-specific regulation will therefore require substrate-selective enzyme targeting or epitope-directed delivery systems.

In summary, targeting aberrant AGP glycosylation currently constitutes a theoretical therapeutic framework rather than an established opportunity; whether such interventions can suppress tumor progression, alleviate inflammation, or improve clinical outcomes awaits further causal and translational investigation.

### 5.3. Major Challenges in the Clinical Translation of AGP Glycosylation

Despite substantial advances in AGP glycosylation research, its translation into clinical practice still faces three major challenges. First, analytical limitations: glycosylation is characterized by substantial structural complexity and pronounced interindividual heterogeneity, while current analytical methods still have notable limitations in sensitivity, specificity, and procedural standardization. A unified and broadly applicable workflow for routine clinical testing has yet to be established. Second, insufficient evidence for causal mechanisms: the upstream and downstream causal relationships between AGP glycosylation dysregulation and disease pathogenesis remain incompletely understood. Mechanistic studies are still fragmented, and a comprehensive and systematic molecular regulatory network governing AGP glycosylation has not yet been established. In addition, large-scale, multicenter clinical cohort data remain insufficient to substantiate the reported associations. Third, the lack of an established framework for clinical translation: unified, reproducible, and standardized analytical platforms are still lacking, and the comparability of experimental data across studies remains limited, substantially constraining the translation of AGP glycosylation biomarkers from laboratory research into routine clinical diagnosis and management.

In addition, different laboratories employ different sample-preparation protocols and mass-spectrometric platforms, resulting in inconsistent quantitative measurements of AGP glycoforms even for the same population samples. Some studies have even reported conflicting findings. Such interlaboratory methodological variability represents a further practical barrier to the clinical translation of AGP glycosylation biomarkers.

### 5.4. Future Research Priorities for Clinical Translation

To address the three major challenges outlined above, future studies should focus on three principal directions to facilitate the translation of AGP glycosylation research from basic investigation to clinical application. First, standardized quantitative glycomic analysis should be established. High-throughput and highly specific methods for quantitative analysis of AGP glycosylation should be developed, together with harmonized operating procedures and evaluation criteria, to establish standardized analytical platforms suitable for clinical samples and minimize methodological heterogeneity. Second, multi-omic integration should be used to elucidate the molecular regulatory mechanisms of AGP glycosylation. Genomic, transcriptomic, and proteomic datasets should be integrated to systematically characterize the multilayered regulatory network governing AGP glycosylation, including the coordinated contributions of glycosyltransferases, cellular signaling pathways, and tissue microenvironments, and to clarify the mechanisms underlying the formation of disease-specific glycoforms. Artificial intelligence (AI)-based algorithms may further be applied to identify disease-specific AGP glycosylation “molecular fingerprints”, thereby supporting precise disease stratification and prediction of therapeutic response. Further studies should also establish the causal relationships between aberrant glycosylation and disease pathogenesis and address the current gaps in mechanistic evidence. Third, interdisciplinary collaboration should be strengthened to advance clinical implementation. Coordinated efforts across glycobiology, laboratory medicine, clinical medicine, and pharmacology will be required to promote the clinical application of AGP glycosylation biomarkers in early disease screening, inflammation monitoring, and prognostic assessment. In parallel, the development of novel therapeutics targeting AGP glycosylation pathways should be pursued, with the ultimate goal of linking fundamental discoveries to clinical decision-making and advancing precision medicine.

## 6. Conclusions

Site-specific glycoform reprogramming of AGP generates disease-specific molecular signatures, metaphorically referred to in this review as a “glycan code”. Aberrant AGP glycosylation patterns represent characteristic molecular features shared across multiple pathological conditions, including inflammation, cancer, and autoimmune diseases, and have been linked to key regulatory processes throughout disease progression, although whether they contribute causally to disease development or progression remains to be established.

In summary, the rapid advancement of glycomic technologies in recent years, particularly highly sensitive mass spectrometry, has enabled precise characterization of the fine structural features of site-specific AGP glycosylation, established its potential as a novel biomarker, and provided an important foundation for the clinical translation of glycobiological discoveries. With continued improvement in standardized analytical systems and increasingly close interdisciplinary collaboration, AGP glycosylation research is expected to provide strong theoretical and technical support for elucidating the mechanisms underlying disease heterogeneity and developing individualized diagnostic and therapeutic strategies.

AGP glycosylation varies across inflammatory, malignant, and autoimmune conditions. These disease-associated features are promising candidates for biomarker research, but their specificity and incremental clinical value require independent validation. Functional studies support glycan-dependent effects in selected experimental settings; they do not establish that the glycosylation differences observed in patients are causal drivers of disease progression.

## Figures and Tables

**Table 1:** Glycan features of the five N-glycosylation sites of AGP (based on Imre et al., 2005 [11]) ^a^.

Site (Asn Position)	Major Glycoform	Branching Feature	Sialylation	Fucosylation
I (Asn-15)	Tri-antennary complex type (TriS3 as the dominant form)	Low branching degree, predominantly tri-antennary, with minor bi-antennary and tetra-antennary forms	S2, S3	F1
II (Asn-38)	Tri-antennary complex type (TriS3 as the dominant form)	Low branching degree, predominantly tri-antennary, with minor bi-antennary forms	S2, S3	F1
III (Asn-54)	Tetra-antennary complex type (TetraS3, TetraS4 as the dominant forms)	High branching degree, predominantly tetra-antennary, with minor tri-antennary and bi-antennary forms	S2–S4	F1
IV (Asn-75)	Tetra-antennary complex type (TetraS3, TetraS4 as the dominant forms)	Predominantly tetra-antennary; additional LacNAc-containing forms reported, without definitive assignment of extra antennae	S1–S4	F1, F2
V (Asn-85)	Tetra-antennary complex type (TetraS4 as the dominant form)	Predominantly tetra-antennary; additional LacNAc-containing forms reported, without definitive assignment of extra antennae	S2–S4	F1, F2

Note: ^a^ The prefixes bi-, tri-, tetra-, penta- and hexa- follow the nomenclature used in the source study and denote the number of N-acetyllactosamine (Gal–GlcNAc) units in the glycan rather than the number of antennae. Glycans designated as penta- or hexa-antennary may therefore reflect either an increased number of antennae or poly-LacNAc extensions of the existing antennae; these two structural alternatives cannot be distinguished on the basis of the mass-spectrometric data reported in [11]. The number following S indicates the number of sialic acid (NeuAc) residues, whereas the number following F indicates the number of fucose residues.

**Table 2:** Relative abundances of N-linked glycans with different antennarity and fucosylation states in plasma AGP from patients with colorectal cancer and healthy controls.

Glycan Category	Specific Glycan Subtype	Relative Abundance (%)		*p* Value	Mechanistic Association (Original Study Conclusions)
		Healthy Individuals (*n* = 35)	Colorectal Cancer Patients (*n* = 67)		
Bi-antennary glycans	Fucose-free (Fuc = 0)	36.12 ± 15.52	42.07 ± 14.03	n.s.	Not involved in cancer-associated glycosylation remodeling; stable expression with no statistical difference between groups
Tri-antennary glycans	Fucose-free (Fuc = 0)	37.44 ± 12.57	25.84 ± 9.76	<0.001	Serves as a “structural precursor” for fucosylation, converting to fucosylated forms, showing a “substrate consumption” characteristic
	Monofucosylated (Fuc = 1)	8.26 ± 3.63	14.60 ± 5.42	<0.001	Core cancer-associated glycoform, major component of FUCAGP, closely related to tumor invasiveness and poor prognosis
	Difucosylated (Fuc = 2)	1.40 ± 1.05	2.10 ± 1.06	<0.001	Assists monofucosylated forms in enhancing tumor cell adhesion and promoting tumor progression
	Total fucosylated (Fuc = 1–2)	9.66 ± 3.02	16.69 ± 5.42	<0.001	Overall upregulation of tri-antennary glycan fucosylation, a core signal for cancer diagnosis
Tetra-antennary glycans	Fucose-free (Fuc = 0)	7.95 ± 4.15	5.41 ± 2.19	<0.001	Similar to fucose-free tri-antennary glycans, serves as a precursor for fucosylation; expression decreases with cancer progression
	Monofucosylated (Fuc = 1)	1.42 ± 1.16	2.32 ± 1.30	<0.001	Cooperates with tri-antennary fucosylated forms to form the FUCAGP marker system, improving diagnostic specificity
	Difucosylated (Fuc = 2)	0.57 ± 0.50	0.66 ± 0.57	n.s.	Extremely low expression, limited impact on overall fucosylation levels, no independent diagnostic value
	Trifucosylated (Fuc = 3)	0.29 ± 0.28	0.34 ± 0.36	n.s.	Rarely expressed, no obvious association with cancer progression; excluded as a prognostic marker
	Total fucosylated (Fuc = 1–3)	2.28 ± 1.65	3.32 ± 1.82	<0.01	Enhanced fucosylation of tetra-antennary glycans, further consolidating the tumor specificity of FUCAGP
Core Marker Summary	FUCAGP (Fucosylated tri- + tetra-antennary)	18.68 ± 6.33	23.02 ± 5.70	<0.001	Core prognostic indicator confirmed in the original study; dramatically elevated in patients with poor prognosis, directly related to tumor recurrence, with high diagnostic specificity
Structural Control Reference	Fucose-free tri- + tetra-antennary (Fuc = 0)	62.12 ± 17.09	55.69 ± 14.88	n.s.	Confirms that cancer-associated glycosylation changes are limited to fucosylation modification rather than the total number of antennary structures, excluding structural remodeling interference

Table 2 was compiled from data reported by Asao et al., 2013 [47], and compares the relative abundances of plasma AGP N-glycans with different antennarity and fucosylation states between healthy individuals and patients with colorectal cancer. Glycans were quantified by mass spectrometry using AGPAS software and are expressed as percentages of the total glycan peak area (mean ± standard deviation). Differences between groups were assessed in the original study; in the *p* Value column, n.s. denotes a difference that was not statistically significant (as reported by Asao et al.), whereas <0.01 and <0.001 denote significant differences at the respective levels. AGP N-glycans share the conserved Man_3_GlcNAc_2_ N-glycan core. FUCAGP, defined as the sum of the relative abundances of fucosylated tri- and tetra-antennary glycans, was evaluated in the original study as a candidate diagnostic and prognostic indicator for colorectal cancer. The data indicate that colorectal cancer-associated AGP glycosylation remodeling is characterized primarily by alterations in fucosylation rather than by a major shift in overall glycan antennarity.

**Table 3:** Core features of AGP glycosylation across different pathological conditions.

Pathological Type		Analytical Basis	Branching Characteristics	Fucosylation Characteristics	Sialylation Characteristics	Functions and Clinical Significance
Acute Inflammation		Mixed: site-specific (glycopeptide) [37]; protein-level (monosaccharide composition and lectin) [38]; mechanistic, non-AGP system [39,40];	Increased proportion of bi-antennary glycans; reduced tri- and tetra-antennary branching [37]	Increased α1,3-fucosylation [37]	Marked increase in α2,6-linked sialylation [38]	Blocks C1q binding to inhibit excessive activation of the classical complement pathway [39]; engages inhibitory Siglec-9 signaling to restrain inflammatory amplification and excessive immune activation [40]
Chronic Inflammation		Protein-level glycoform (Con A crossed affinoimmuno-electrophoresis) [41]	Glycosylation fluctuations synchronized with persistent inflammatory burden [41]	Glycoform alterations associated with concomitant chronic inflammation [41]	Not specifically reported	Provides a quantitative readout of persistent inflammatory burden; candidate biomarker for longitudinal monitoring of chronic inflammation [41]
Autoimmune Diseases		Mixed: released glycan (PNGase F/HPAEC) [63]; PNGase F/HPAEC-PAD with functional binding assay [57]); protein-level glycoform (Con A affinoimmuno-electrophoresis [66]; AAL lectin ELISA [64,65]); Con A reactivity and monosaccharide composition [68]); computational (molecular-dynamics structural modelling) [10]; review [3,27,69]	Marked increase in glycan branching [63]; glycoform composition shifts with disease duration and activity [66]	Overall increase in fucosylation [57,63,64,65]; magnitude correlates positively with inflammatory severity [65]; pronounced sex-related difference—higher baseline in men, DAS28 association reported only in male patients [64]	Predominantly complex-type N-glycans with terminal sialylation; α2,3-/α2,6-linkage variation influences protein stability and immunoregulatory activity [3]	Modulates binding to vascular endothelial E-selectin via the sLe^X^ epitope and influences leukocyte adhesion [10,27,57]; sialylation/fucosylation shifts modulate E-selectin binding [3,57,68]; may activate NF-κB/JAK-STAT pathways [27,69]; fucosylation dynamics track therapeutic response [65]
Tumor	Lung Cancer	Protein-level glycoform (lectin–antibody EIA, AAL) [43]	Not specifically reported	Serum α1,3-fucosylated AGP (fAGP) increases progressively with disease progression [43]	Not specifically reported	Associated with response to immunotherapy and long-term survival; prognostic biomarker [43]
	Breast Cancer	Protein-level glycoform (enzyme-linked lectin assay, ELLA) [44]	Tumor-stage-dependent variation [44]	Tumor-stage-dependent variation in fucosylation and high-mannose glycans; lowest in stage I [44]	Tumor-stage-dependent variation in sialylation [44]	Potential value for adjunctive early diagnosis [44]
	Hepatocellular Carcinoma	Released glycan (PNGase F/MALDI-MS profiling) [45]	Distinctive tetra-antennary N-glycan structure [45]	Trifucosylated tetra-antennary N-glycans (triFc_AGP) [45]	Not specifically reported	Distinguishes HCC patients from healthy individuals and patients with cirrhosis; supports disease stratification [45]
	Malignant Melanoma	Released glycan (PNGase F/HILIC-MS/MS) [46]	Selected tri- and tetra-antennary glycans increased [46]	Selected fucosylated glycans increased; non-fucosylated glycans decreased [46]	Increased proportion of α2,6-linked sialic acid [46]	Potential serum biomarker to support clinical diagnosis [46]
	Colorectal Cancer	Released glycan (PNGase F/MS with AGPAS) [47]	No major shift in overall glycan antennarity [47]	Alterations primarily in fucosylation; FUCAGP elevated [47]	Not specifically reported	Candidate diagnostic and prognostic indicator; elevated in poor prognosis and tumor recurrence [47]

Findings are grouped according to the analytical basis on which they were reported. “Released glycan” denotes analysis of N-glycans enzymatically released from total AGP (or total plasma glycoprotein), which reports the aggregate glycan profile and does not resolve individual glycosylation sites. “Site-specific” denotes glycopeptide- or site-resolved analysis that assigns glycans to individual N-glycosylation sits of AGP. “Protein-level” denotes analysis of intact AGP glycoforms resolved by lectin affinity, electrophoretic, or chromatographic methods, which distinguishes glycoform populations but does not assign glycans to specific sites. These levels of evidence are not interchangeable: an alteration detected at the released-glycan or protein level reflects the summed contribution of all five N-glycosylation sites and does not identify the contributing site(s). Conclusions regarding site-specific remodeling are drawn only where site-resolved evidence is available. References [39,40] report mechanistic studies performed in non-AGP experimental systems and are cited for mechanistic context only. Reference [10] reports molecular-dynamics structural modelling and is therefore classified as computational rather than as experimental glycomic evidence. References [15,42] report site-specific glycopeptide analyses that are discussed in Section 3.1.2 as contextual variables for interpreting AGP glycosylation data; they are not entered into the Chronic Inflammation row. Abbreviations: AAL, Aleuria aurantia lectin; CAIE, crossed affinoimmunoelectrophoresis; EIA, enzyme immunoassay; ELLA, enzyme-linked lectin assay; HILIC, hydrophilic interaction chromatography; HPAEC, high-pH anion-exchange chromatography; HCC, hepatocellular carcinoma; PNGase F, peptide-N-glycosidase F; sLeX, sialyl-Lewis X; FUCAGP, fucosylated tri- and tetra-antennary AGP glycans; triFc_AGP, trifucosylated tetra-antennary AGP glycan.

## Data Availability

No new data were created or analyzed in this study. Data sharing is not applicable to this article.
